# *XRCC4* rs28360071 intronic variant is associated with increased risk for infant acute lymphoblastic leukemia with *KMT2A* rearrangements

**DOI:** 10.1590/1678-4685-GMB-2020-0160

**Published:** 2020-12-02

**Authors:** Orlando Louzada-Neto, Bruno A. Lopes, Gisele D. Brisson, Francianne G. Andrade, Ingrid S. Cezar, Cíntia B. Santos-Rebouças, Rodolpho M. Albano, Maria S. Pombo-de-Oliveira, Ana Rossini

**Affiliations:** 1 Universidade do Estado do Rio de Janeiro, Departamento de Bioquímica, Laboratório de Toxicologia e Biologia Molecular, Rio de Janeiro, RJ, Brazil.; 2 Centro de Pesquisas, Instituto Nacional do Câncer, Programa de Hematologia-Oncologia Pediátrica, Rio de Janeiro, RJ, Brazil.; 3 Universidade do Estado do Rio de Janeiro, Departamento de Genética, Rio de Janeiro, RJ, Brazil.

**Keywords:** Early age acute leukemia, nonhomologous end-joining, XRCC4, XRCC6, KMT2A

## Abstract

Early age acute leukemia (EAL) shows a high frequency of *KMT2A*-rearrangements (*KMT2A*-r). Previous investigations highlighted double-strand breaks arising from maternal exposure to xenobiotics during pregnancy as a risk factor for EAL and *KMT2A*-r. In this case-control study, we investigated the relationship between EAL and genetic variants of the nonhomologous end-joining (*XRCC6* rs5751129, *XRCC4* rs6869366 and rs28360071), since they might affect DNA repair capacity, leading to *KMT2A*-r and leukemogenesis. Samples from 577 individuals (acute lymphoblastic leukemia-ALL, n=164; acute myeloid leukemia-AML, n=113; controls, n=300) were genotyped. No significant association was found for rs5751129 and rs6869366, whereas rs28360071 was associated with an increased risk for ALL with *KMT2A-r* (IIxID: OR - Odds ratio 2.23, CI 1.17-4.25, *p*=0.014). Bone marrow samples from ALL patients showed a higher expression of *XRCC4* compared to AML patients (*p*=0.025). Human Splicing Finder 3.1 predicted that the deleted allele of rs28360071 is potentially associated with the activation of a 5’ cryptic splice site in intron 3 of *XRCC4*. The sequencing of cDNA did not show any differences on the splicing process for the rs28360071 genotypes. Our results suggest that the deleted allele for rs28360071 increases the risk for ALL with *KMT2A-r*, but not by modifying the *XRCC4* expression levels or its structure.

## Introduction

Early age acute leukemia (EAL) here defined as children aged up to 24 months old at the diagnosis) is a heterogeneous group of hematopoietic malignancies that includes neonatal and infant leukemia and is the leading cause of death by neoplastic diseases in this age group (Noone *et al.*, 2018). The etiology of EAL might be related to an interplay between individual genetic susceptibility and exposure to genotoxic agents with potential carcinogenic action (Alexander *et al.*, 2001; Pombo-de-Oliveira and Andrade, 2016). Common somatic aberrations found in EAL are rearrangements involving the *KMT2A* gene (*Lysine K-specific Methyltransferase 2A*, also named *Mixed Lineage Leukemia*, *MLL*) (Felix, 2001). Since the KMT2A protein plays an essential role in the positive regulation of gene expression during human fetal development and hematopoiesis, (*KMT2A*-r - *KMT2A* rearranged) promotes both infant acute lymphoblastic leukemia (ALL - Acute lymphoblastic leukemia) and infant acute myeloid leukemia (AML) (Slany, 2009). **‬‬‬‬‬‬‬‬‬‬‬‬‬‬‬‬‬‬‬‬**

Translocations can occur after the generation of double-strand breaks (DSB - Double strand break) along one or more chromosomes and incorrect chromosomal rejoining. Several studies have highlighted that *KMT2A*-r generation occurs *in-utero* (Greaves and Wiemels, 2003). The association between EAL, *KMT2A*-r and maternal exposure to substances that can cross the placental barrier and cause DSBs in fetal DNA has been reported in epidemiological studies (Hutt and Kalf, 1996; Alexander *et al.*, 2001; Pombo-de-Oliveira and Koifman, 2006). In the absence of proper DNA repair mechanisms, the maintenance of genomic integrity after exposure to these compounds would be affected, thus increasing the risk of *KMT2A*-r and EAL.

In eukaryotic cells, DSBs are primarily repaired by nonhomologous end-joining (NHEJ), which might occur in two different pathways: canonical NHEJ (c-NHEJ) and alternative NHEJ (a-NHEJ - Alternative non-homologous end-joining) (Deriano and Roth, 2013). C-NHEJ starts with the recognition of the extremities of DSBs by the heterodimer formed by Ku70 and Ku80 proteins, encoded by the genes *XRCC6* and *XRCC5*, respectively. Another central element involved in c-NHEJ is *XRCC4 (*X-Ray Cross Complementing 4), which encodes a core harbor protein that activates and leads DNA Ligase IV (LIG4) to the damage site to rejoin the strands (Lieber, 2008). However, a-NHEJ occurs in the absence of some c-NHEJ (Canonical non-homologous end-joining) central elements, including Ku70/80 and XRCC4 proteins, and it is considered an error-prone and unfaithful repair mechanism, potentially leading to the formation of deletions and chromosome translocations (Kabotyanski *et al.*, 1998; Simsek and Jasin, 2010).

The aim of the present study was to conduct the first investigation focusing on the relationship between *XRCC6* rs5751129, *XRCC4* rs6869366, and *XRCC4* rs28360071 polymorphisms and the risk for developing EAL with *KMT2A*-r. The rs5751129 is a single nucleotide polymorphism (SNP) within the promoter region of *XRCC6,* which was previously proved to modulate the risk of other cancer types, probably by lowering the mRNA expression levels (Jia *et al.*, 2015). The *XRCC4* rs6869366 is also a SNP located at the promoter region, whereas rs28360071 represents a 30 bp deletion/insertion within intron 3 of *XRCC4*. The implications of these later variants remain to be clarified. However, both are associated with increased risk for several cancer types, including acute childhood leukemia (Wu *et al.*, 2010; Shao *et al*., 2013; Jin *et al*., 2019; Gupta *et al*., 2020).

## Material and Methods

### Subjects

In total, 277 samples (164 ALL and 113 AML cases) from Brazilian infants and toddlers (ages <24 months at the diagnosis of acute leukemia) were included in this study. These samples were obtained from the Brazilian Collaborative Study Group of Infant Acute Leukemia studies (2000-2013) (Pieters, 2009). DNA samples from the umbilical cord blood of healthy subjects (n=300) were genotyped as controls. EAL encompasses a group of leukemias that affect neonates (≤31 days), infants (≤12 months), and toddlers (up to 24 months). Our EAL cohort was composed of infant-ALL, which is defined when leukemia diagnosis occurred in children ≤12 months-old, based on its distinct clinical characteristics, such as pro-B immunophetotype (CD10^neg^) as well as high frequency of *KMT2A-r*. On the other hand, the time-frame for AML inclusion was extended to ≤ 24 months of age at diagnosis, given that *KMT2A-r* are more evenly distributed between infants and toddlers with this leukemia subtype (Pieters, 2009; Creutzig *et al.*, 2012; Emerenciano *et al.*, 2013). In the present study, the exclusion criteria for cases were T-cell precursor ALL, non-hematological or hematological malignancy syndromes with genetic alterations, such as Noonan Syndrome and Down Syndrome, and Myelodysplasic Syndrome. EAL patients with insufficient or low DNA quality samples and without parental consent were excluded from the study. 

The diagnosis of acute leukemia subtypes was applied throughout bone marrow morphology, immunophenotyping, and molecular-cytogenetic alterations, according to World Health Organization criteria (Arber *et al.*, 2016).

The race/ethnicity was defined according to the Instituto Brasileiro de Geografia e Estatística (IBGE), which gathers subjects according to skin color self-definition. The subjects were categorized into whites or non-whites. The group of non-whites comprised self-categorized brown or African-descent subjects. 

The present study was approved by the Ethics and Scientific Committee of the Instituto Nacional de Câncer (CEP-CONEP # 626.268; April 24^th^, 2014).

### KMT2A status analysis

Bone marrow RNA extraction, cDNA synthesis, and quantitative polymerase chain reaction (qPCR) were performed as described elsewhere (Lopes *et al.*, 2015). Briefly, RNA was extracted from bone marrow samples using TRIzol reagent (Invitrogen, CA, USA) according to the manufacturer’s instructions. Following DNase treatment (Invitrogen, CA, USA), cDNA was synthesized using SuperScript III Reverse Transcriptase (Invitrogen, CA, USA) with 3 μg of purified RNA as a template. *KMT2A* status was evaluated by reverse transcription-PCR assays for the detection of the main chimeric transcripts (fusion between *KMT2A* and *AFF1, MLLT3, MLLT1, MLLT10,* or *MLLT4*), and/or by fluorescence *in situ* hybridization (Vysis LSI MLL Dual Color, Break Apart Rearrangement Probe, Abbott Laboratories, Abbot Park, IL, USA) as previously described (Emerenciano *et al.*, 2006; Burmeister *et al.*, 2015).

### DNA extraction and genotyping

DNA extraction was performed with the QIAamp DNA Mini kit (Qiagen Inc., Germantown, MD, USA) according to the manufacturer’s protocols. Genotyping was performed by PCR, followed by restriction fragment length polymorphism (RFLP) for *XRCC6* rs5751129 (informally cited as -991 T>C) and *XRCC4* rs6869366 (informally cited as -1394 T>G) according to a previous report (Tsai *et al.*, 2007). Subsequently, the alleles of insertion (139 bp) and deletion (109 bp) of *XRCC4* rs28360071 (informally cited as intron 3 DIP) were detected by 2% agarose gel electrophoresis. 

### Gene expression

We evaluated *XRCC4* mRNA expression in 42 samples (25 ALL and 17 AML) by qPCR using the following primer sequences: 5’- TGGACTGGGACAGTTTCTGA-3’ (forward) and 5’- CTGCTCCTGACAACAATGCT- 3’ (reverse), as previously described (Shao *et al.*, 2014). The glyceraldehyde 3-phosphate dehydrogenase (GAPDH) gene was used for the normalization of *XRCC4* transcript using the 5’-CAACAGCCTCAAGATCATCAGCAA-3’ (forward) and 5’-AGTGATGGCATGGACTGTGGTCAT-3’ (reverse) primers. The experiments were performed in triplicates, and Ct medians were used to determine the relative expression by the 2^-ΔCt^ method (Livak and Schmittgen, 2001).

### Sequencing

For evaluating if the splicing process is different among the three genotypes for rs28360071, a pair of primers on exon 3 (Forward: 5’-GGAGCAGGACCAGCTGATGTAT-3’) and exon 4 (Reverse: 5’-TTTCTGCAATGGTGTCCAAGC-3’) of *XRCC4* cDNA was designed. After that, cDNA samples from five random patients (two carrying II, one carrying ID (Insertion/Deletion. Heterozygous genotype of rs28360071) and two carrying DD (Deletion/Deletion. Homozygous genotype of deletion allele of rs28360071) genotypes) were amplified by PCR. The amplified fragment resulting from the splicing process was expected as a 175 bp fragment (Figure S3b). PCR fragments were purified using Agencourt AMPure XP magnetic beads (Beckman Coulter Company, MA, USA), and sequenced with the BigDye Terminator v3.1 Cycle Sequencing Kit (Thermo Fisher Scientific Inc.) on a 3500 Genetic Analyser (Thermo Fisher Scientific Inc.). Experiments were performed in triplicates. Representative chromatograms are shown in Figure S3.

### **Statistical and *in silico* analysis**

The chi-square test was used for the comparison of sociodemographic frequencies between cases and controls, such as child age, sex, ethnicity/skin color, and *KMT2A* status. Expected genotypes frequencies of the genetic polymorphisms were calculated by Hardy-Weinberg (HW) with GenePop Web version 4.5.1 (http://genepop.curtin.edu.au/). The Odds Ratios (ORs) with 95% confidence intervals (CI) were calculated using chi-square and the Fisher's exact tests. Disease risk was calculated for dominant (wild-type homozygote vs. polymorphic heterozygote + polymorphic homozygote), recessive (wild-type homozygote + heterozygote vs. polymorphic homozygote), codominant (wild-type homozygote vs. heterozygote or wild-type homozygote vs. polymorphic homozygote), and additive (wild-type homozygote vs. heterozygote + two times polymorphic homozygote) models. The Statistical Product and Services Solutions statistical package 22.0 (SPSS Inc., Chicago, IL, USA) was used to calculate odds ratios (ORs) with 95% confidence intervals (CIs) and *p* values while unconditional logistic regression was used to estimate adjusted odds ratios (aORs). For all analyses, the *p* values < 0.05 were considered significant. Bonferroni correction for testing three SNPs was applied to the adjusted *p* value. The Bonferroni-corrected statistical significance level set at *p* values < 0.017 (0.05/3) indicated statistically significant results. The qPCR statistical analysis was performed with GraphPad Prism 5 (GraphPad Software, Inc., California, USA). The comparison among genotypes in relation to *XRCC4* expression was performed using the Kruskal-Wallis tests. The Mann-Whitney test was used for analyses related to *KMT2A* status.

The online bioinformatics tool Human Splicing Finder version 3.1 software http://www.umd.be/HSF3/index.html (HSF, 2019) was applied to predict the potential functional impact of the indel polymorphism at *XRCC4* splicing sites. This software is an international reference that combines twelve different algorithms to identify and predict the effect of a variant on splicing motifs, including the acceptor and donor splice sites, the branch point, and auxiliary sequences known to either enhance or repress splicing.

## Results

### XRCC4 deleted allele rs28360071 increases the risk for ALL in patients with KMT2A-r

The frequency distribution of demography characteristics, childhood leukemia subtypes, and *KMT2A* status are described in Table 1. The children were ≤ 2 years old at the diagnosis of acute leukemia, with a mean age of 13 ± 5.6 months at the time of diagnosis (ALL= 12 ± 7.0 months and AML = 13 ± 5.7 months). The distribution of cases and controls by sex was similar, but regarding to ethnicity, there was a higher number of whites in both groups (p= 0.010). All genotype distribution of polymorphisms was in Hardy-Weinberg equilibrium (Table S1).

**Table 1 -:** The frequency distribution of childhood leukemia, demography and *KMT2A* status, Brazil, 2000-2013.

	Cases n (%)	Controls n (%)	***p* Value**
Acute Leukemia Subtype			
ALL	164 (59.2)	-	-
AML	113 (40.8)	-	
Sex			
Male	147 (53.5)	144 (48.0)	0.211
Female	128 (46.5)	156 (52.0)	
NI	2	0	
Ethnicity			
White	164 (60.3)	136 (49.0)	0.010
Non-white*	108 (39.7)	142 (51.0)	
NI	5	22	
*KMT2A* Status			
*KMT2A*-r	103 (48.1)	-	-
*KMT2A*-WT	111 (51.9)	-	
NI	63	-	
Total	277 (48.0)	300 (52.0)	-

ALL: Acute Lymphoblastic Leukemia; AML: Acute Myeloid Leukemia; NI: No Information available; *KMT2A*-r: *KMT2A* rearranged; *KMT2A*-WT: *KMT2A* wild type.

(*) Comprises Brazilians with genetic admixture and self-defined skin color.

No significant association between the *XRCC6* rs5751129 variant and EAL risk was observed in any analyzed parameter (Table S2). The *XRCC4* 6869366 variant demonstrated a protective effect on *KMT2A*-r only among ALL cases under the dominant (OR = 0.30; CI = 0.09-1.00; *p* = 0.040) and additive (OR = 0.30; CI = 0.10-0.95; *p* = 0.040) models (Table S3). However, the statistical significance of those associations was lost by adjusting OR by skin color and sex (dominant model: aOR = 0.30; CI = 0.08-1.01; *p* = 0.054 and additive model: aOR = 0.42; CI = 0.01-1.11; *p* = 0.050). However, for the *XRCC4* rs28360071 an increased risk for ALL with *KMT2A*-r was associated with the heterozygous genotype (Codominant model; homozygous for insertion II vs. heterozygous ID: OR = 2.00; CI = 1.10-3.80; *p* = 0.039), even when adjusted (aOR = 2.23; CI = 1.17-4.25; *p* = 0.014) (Table 2). The results remained statistically significant after Bonferroni’s correction (p = 0.014 x 3 = 0.042).

**Table 2:** Genotype frequencies for *XRCC4* rs28360071 in EAL subtypes and *KMT2A* status.

***XRCC4* rs28360071**	Control n (%)	ALL n (%)		AML n (%)	
II	87 (30.3)	42 (26.9)		35 (31.5)	
ID	134 (46.7)	85 (54.5)		50 (45.0)	
DD	66 (23.0)	29 (18.6)		26 (23.4)	
Genetic Model	*p* OR (CI)	*p* aOR (CI)	*p* OR (CI)	*p* aOR (CI)
Dominant	0.510 1.18 (0.80-1.82)	0.250 1.07 (0.75-1.55)	0.810 0.95 (0.60-1.52)	0.911 0.97 (0.59-1.58)
Recessive	0.330 0.80 (0.50-1.25)	0.247 0.90 (0.60-1.30)	1.000 1.02 (0.61-1.72)	0.830 0.94 (0.55-1.61)
II vs. ID	0.251 1.31 (0.83-2.10)	0.103 1.14 (0.80-1.70)	0.800 0.93 (0.60-1.54)	0.992 0.99 (0.58-1.68)
II vs. DD	0.773 0.91 (0.51-1.61)	0.931 0.94 (0.60-1.51)	1.000 1.00 (0.54-1.80)	0.837 0.93 (0.50-1.74)
Additive	0.671 1.11 (0.73-1.70)	0.379 0.82 (0.53-1.26)	0.910 0.95 (0.60-1.50)	0.870 1.04 (0.65-1.66)
***KMT2A*-WT**				
***XRCC4* rs28360071**	Control n (%)	ALL n (%)		AML n (%)	
II	87 (30.3)	19 (30.6)		11 (25.0)	
ID	134 (46.7)	28 (45.2)		21 (47.7)	
DD	66 (23.0)	15 (24.2)		12 (27.3)	
Genetic Model	*p* OR (CI)	*p* aOR (CI)	*p* OR (CI)	*p* aOR (CI)
Dominant	1.000 1.00 (0.54-1.80)	0.874 0.95 (0.51-1.74)	0.600 1.30 (0.63-2.70)	0.308 0.67 (0.31-1.44)
Recessive	0.900 1.10 (0.60-2.03)	0.937 0.97 (0.50-1.86)	0.600 1.30 (0.61-2.60)	5.053 0.83 (0.40-1.74)
II vs. ID	1.000 1.00 (0.50-1.90)	0.913 0.96 (0.50-1.85)	0.700 1.25 (0.60-2.70)	0.357 0.68 (0.30-1.54)
II vs. DD	1.000 1.04 (0.50-2.20)	0.850 0.92 (0.43-2.00	0.501 1.45 (0.60-3.50)	0.373 0.66 (0.26-1.64)
Additive	1.000 1.00 (0.60-1.80)	0.850 0.94 (0.52-1.69)	0.502 1.35 (0.70-2.70)	0.279 0.66 (0.31-1.39)
***KMT2A*-r**				
***XRCC4* rs28360071**	Control n (%)	ALL n (%)		AML n (%)	
II	87 (30.3)	16 (20.8)		10 (43.5)	
ID	134 (46.7)	49 (63.6)		9 (39.1)	
DD	66 (23.0)	12 (15.6)		4 (17.4)	
Genetic Model	*p* OR (CI)	*p* aOR (CI)	*p* OR (CI)	*p* aOR (CI)
Dominant	0.120 1.70 (0.91-3.04	0.057 1.81 (0.98-3.37)	0.242 0.60 (0.24-1.34)	0.333 0.64 (0.26-1.57)
Recessive	0.210 0.62 (0.32-1.21)	0.150 0.60 (0.30-1.19)	0.800 0.70 (0.23-2.15)	0.602 0.74 (0.24-2.28)
II vs. ID	0.039 2.00 (1.10-3.80)	0.014 2.23 (1.17-4.25)	0.331 0.60 (0.23-1.50)	0.434 0.67 (0.25-1.79)
II vs. DD	1.000 1.00 (0.44-2.23)	0.900 1.06 (0.46-2.45)	0.400 0.53 (0.16-1.80	0.393 0.58 (0.16-2.01)
Additive	0.210 1.50 (0.82-2.70)	0.115 0.61 (0.33-1.12)	0.171 0.60 (0.25-1.30)	0.276 1.60 (0.68-3.76)

ALL: Acute Lymphoblastic Leukemia; AML: Acute Myeloid Leukemia; OR: Odds ratio; aOR: Odds ratio adjusted by skin color and sex; *KMT2A*-WT: *KMT2A* wild type; *KMT2A*-r: *KMT2A* rearranged; II: homozygous for insertion; ID: heterozygous; DD: homozygous for deletion.

### There is a higher expression of XRCC4 in ALL patients

**Figure 1 -:**
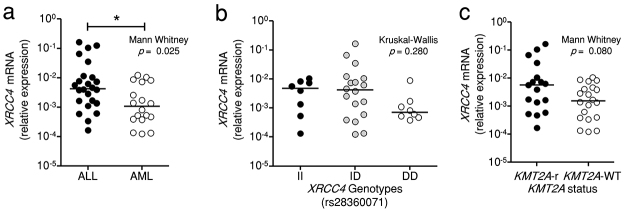
Differential levels of *XRCC4* mRNA in EAL bone marrow samples. **(a)** Comparison of the expression levels of *XRCC4* in acute lymphoblastic leukemia (ALL) and acute myeloid leukemia (AML). **(b)** Differential levels of *XRCC4* according to the genotypes for the *XRCC4* rs28360071 polymorphism. **(c)** Comparison of *XRCC4* expression levels in patients harboring *KMT2A* rearranged (*KMT2A*-r) and *KMT2A* wild type (*KMT2A*-WT). Experiments were performed in triplicates. Horizontal bars indicate median values.

Due to the observed association of *XRCC4* and ALL, *XRCC4* mRNA expression levels in the bone marrow of patients was evaluated by qPCR. A higher expression of *XRCC4* was found in ALL compared to AML (*p* = 0.025) (Fig. 1a). No differential expression was observed according to the three genotypes for the *XRCC4* rs28360071 polymorphism (Fig. 1b), even when the data were stratified by *KMT2A* status. However, *KMT2A*-r samples seem to show slightly higher *XRCC4* expression than that in *KMT2A* wild type (*KMT2A-*WT - *KMT2A* wild type) samples (*p* = 0.080) (Fig. 1c). Moreover, no differential expression was observed according to either genotypes or *KMT2A* status in both ALL and AML (Figure S1).

### The splicing process of XRCC4 could be altered due to rs28360071

*In silico* analysis performed by HSF predicted three changes on intron 3, caused by the deletion of 30 base pairs and activation of an intronic cryptic donor site, an alteration of a splicing silencer site, and the creation of a splicing enhancer site. From those changes, the former was interpreted by the software as having a potential impact on splicing with a high score (Figure S2). We have performed direct sequencing of *XRCC4* in bone marrow cDNA samples, and no structural alterations in cDNA lengths were observed, regardless of the *XRCC4* rs28360071 genotype status (Figure S3). 

## Discussion

In the present study, the correlation between genetic polymorphisms in NHEJ genes and the risk for EAL was evaluated. No correlation was found between the overall risk for EAL with *KMT2A-*WT and *XRCC4* (rs6869366 and rs28360071) or *XRCC6* (rs5751129) variants included here. 

The *XRCC6* rs5751129 SNP is associated with decreased transcript and protein expression levels (Hsu *et al.*, 2013). Individuals carrying the *C allele had lower *XRCC6* mRNA expression (Chang *et al.*, 2012; Hsu *et al.*, 2013). Regarding acute childhood leukemia (0-18 years old), patients carrying the heterozygous genotype had a significantly increased risk of developing the disease (Pei *et al.*, 2013). In the present work, no association between rs5751129 and EAL was found, regardless *KMT2A* status.

Wu and colleagues found an increased risk of leukemia related to both *XRCC4* variants among Taiwanese children (Wu *et al.*, 2010). These authors found that *G and deleted alleles of *XRCC4* rs6869366 and rs28360071, respectively, are associated with a higher risk for childhood leukemia. The present study demonstrates that the *T allele of *XRCC4* rs6869366 has a protective effect against ALL, although the association was not significant after p-value adjustment. Another study showed that *XRCC4* variants are associated with an increased risk for ALL, particularly in ALL cases with chromosomal translocations and hyperdiploidy, both alone and in conjunction with exposure to ionizing radiation (Chokkalingam *et al.*, 2011). 

Here, an increased risk for ALL with *KMT2A*-r was found to be associated with the ID genotype of *XRCC4* rs28360071. These data suggest an influence of the 30-bp deletion allele over the XRCC4 protein or its repair. Other studies have also shown significant associations between *XRCC4* variants and childhood ALL with *ETV6-RUNX1* [t(12; 21)] translocations (Chokkalingam *et al.*, 2011). To examine whether the deletion allele could be related to gene expression levels, *XRCC4* mRNA expression was assessed by qPCR, showing higher expression in ALL than in AML samples. Although not statistically significant, *KMT2A*-r samples showed higher gene expression than that of *KMT2A*-WT samples. Chiou and colleagues studied the expression of the genes related to NHEJ in samples from children with pediatric ALL (Chiou *et al.*, 2007). These studies also showed an increased expression of *XRCC4* mRNA. We did not find any variation of *XRCC4* expression associated with *XRCC4* rs28360071 genotypes, suggesting that the deletion allele could influence the risk for EAL by another mechanism. In this sense, the use of an *in silico* tool pointed out that the deleted allele could potentially implicate *XRCC4* splicing, through activation of a cryptic donor site on intron 3. Cryptic splice sites are dormant or less used splicing sites found both in exons and introns that can be activated by mutations. When activated, these sites could be efficiently used by the splicing machinery, possibly resulting in genetic disorders (Buratti *et al.*, 2007; Wang and Cooper, 2007). In the presence of the 30 bp deletion in intron 3 of *XRCC4*, the activation of a cryptic donor splicing site may lead to preferential use of this donor site in detriment of the original one. As a consequence, this event can lead to the inclusion of part of intron 3 into the mature mRNA, which could cause protein structure modifications. 

Additionally, the defective mRNA may also include a premature stop codon, which could even deflagrate degradation of the mRNA before translation by nonsense-mediated decay, as previously reported (Nicholson and Muhlemann, 2010; Santos-Rebouças *et al.*, 2011). As no changes in the splicing process were observed by *XRCC4* cDNA sequencing, we hypothesize that the influence of the rs28360071 polymorphism on the development of EAL might occur by another biological mechanism. We are not able to determine the impact of this polymorphism on the biology of the protein and how it implicates on NHEJ repair capacity with the data we showed here. Future studies are needed to investigate the molecular mechanisms underlying the phenotypic impact caused by rs28360071.

The present results are not similar to those reported by other groups studying pediatric acute leukemia. This discrepancy is likely due to the characteristic of the study population since the present work includes a unique series of children up to 24 months old, and most studies regarding childhood leukemia include children up to 18 years old. The disease development of EAL is related to a prenatal origin, resulting from *in utero* exposure to DNA damage agents. In contrast, the disease among older children can have a different etiology, including the acquisition of additional genetic aberrations during childhood. Since other studies do not analyze ALL and AML separately, a comparison of the results between studies should be performed carefully. Indeed, we must consider that the Brazilian population has unique miscegenation profile and is one of the most heterogeneous populations worldwide (Parra *et al.*, 2003).

Herein, we assessed the influence of genetic polymorphisms in central elements of c-NHEJ on the development of EAL and *KMT2A*-r. Our hypothesis was based on the fact that maternal exposure to xenobiotic compounds generates DSBs on DNA of fetal hematological stem cells. Once DNA repair is not appropriate, chromosomal translocations could occur within *KMT2A*, thus driving and sustaining *in utero* leukemogenesis. 

To the best of our knowledge, this study is the first to report polymorphisms in the NHEJ repair system and *KMT2A*-r in EAL. Although the risk for EAL was not related to *XRCC6* rs5751129 or *XRCC4* rs6869366 variants, the 30-bp deletion within the third intron of *XRCC4* influenced the risk of ALL with *KMT2A-*r cases. Additional studies are necessary to evaluate the functional impact of these variants in the biology of leukemia.

## Supplementary material

The following online material is available for this article:

Table S1 - Allele and genotype frequencies of *XRCC6* rs5751129, *XRCC4* rs6869366 and *XRCC4* rs28360071 in cases and controls.

Table S2 - Genotype frequencies of *XRCC6* rs5751129 in EAL subtypes and *KMT2A* status.

Table S3 - Genotype frequencies of *XRCC4* rs6869366 in EAL subtypes and *KMT2A* status.

Figure S1 - Differential levels of *XRCC4* mRNA in EAL bone marrow samples.

Figure S2 -
*In silico* predictions for *XRCC4* rs28360071 polymorphism (NM_022550.3:c.315+31090del30).

Figure S3 - Sequencing of bone marrow *XRCC4* cDNA of patients with EAL.
