# Supplementary material for: XRCC4 rs28360071 intronic variant is associated with increased risk for infant acute lymphoblastic leukemia with KMT2A rearrangements
Source: Genet Mol Biol. 2020 Dec 2;43(4):e20200160. doi: 10.1590/1678-4685-GMB-2020-0160 (PMC7734917; doi:10.1590/1678-4685-GMB-2020-0160)
Supplement: Table S1 - [file 1415-4757-GMB-43-4-e20200160-s1.pdf]

https://doi.org/10.1590/1678-4685-GMB-2020-0160

Supplementary Material to "*XRCC4* rs28360071 intronic variant is associated with increased risk for infant acute lymphoblastic leukemia with *KMT2A* rearrangements"

Table S1 – Allele and genotype frequencies of *XRCC6* rs5751129, *XRCC4* rs6869366 and *XRCC4* rs28360071 in cases and controls.

		Controls	Cases
		n (%)	n (%)
XRCC6 rs5751129			
	T	259 (63.2)	369 (63.2)
	C	151 (36.8)	215 (36.8)
	TT	80 (39.0)	118 (40.5)
	TC	99 (48.2)	133 (45.5)
	CC	26 (12.8)	41 (14.0)
	p Value	0.710	0.660
XRCC4 rs6869366			
	T	541 (92.0)	361 (94.5)
	G	47 (8.0)	21 (5.5)
	TT	249 (84.7)	172 (90.0)
	TG	43 (14.6)	17 (8.9)
	GG	2 (0.7)	2 (1.1)
	p Value	0.700	0.100
XRCC4 rs28360071			
	I	597 (53.9)	289 (54.1)
	D	511 (46.1)	245 (45.9)
	II	164 (29.6)	77 (28.8)
	ID	269 (48.5)	135 (50.6)
	DD	121 (21.8)	55 (20.6)
	p Value	0.340	0.805

p Value: Hardy-Weinberg equilibrium.
