# Supplementary material for: XRCC4 rs28360071 intronic variant is associated with increased risk for infant acute lymphoblastic leukemia with KMT2A rearrangements
Source: Genet Mol Biol. 2020 Dec 2;43(4):e20200160. doi: 10.1590/1678-4685-GMB-2020-0160 (PMC7734917; doi:10.1590/1678-4685-GMB-2020-0160)
Supplement: Table S2 - [file 1415-4757-GMB-43-4-e20200160-s2.pdf]

Genetics and Molecular Biology
https://doi.org/10.1590/1678-4685-GMB-2020-0160

Supplementary Material to "*XRCC4* rs28360071 intronic variant is associated with increased risk for infant acute lymphoblastic leukemia with *KMT2A* rearrangements"

Table S2 – Genotype frequencies of *XRCC6* rs5751129 in EAL subtypes and *KMT2A* status.

***XRCC6* rs5751129**	**Control n (%)**	**ALL n (%)**		**AML n (%)**	
**TT**	118 (40.4)	42 (35.0)		38 (44.7)	
**TC**	133 (45.5)	58 (48.3)		41 (48.2)	
**CC**	41 (14.0)	20 (16.7)		6 (7.1)	
**Genetic Model**		***p*** **OR (CI)**	***p*** **aOR (CI)**	***p*** **OR (CI)**	***p*** **aOR (CI)**
**Dominant**		0.320<br>1.26 (0.81-2.00)	0.115<br>1.10 (0.75-1.53)	0.532<br>0.84 (0.52-1.40)	0.700<br>0.89 (0.54-1.49)
**Recessive**		0.542<br>1.22 (0.70-2.20)	0.257<br>0.90 (0.53-1.51)	0.095<br>0.50 (0.20-1.14)	0.142<br>0.50 (0.20-1.25)
**TT vs. TC**		0.410<br>1.22 (0.80-2.00)	0.211<br>1.10 (0.75-1.62)	0.900<br>1.00 (0.60-1.60)	0.984<br>1.00 (0.59-1.70)
**TT vs. CC**		0.402<br>1.40 (0.72-2.60)	0.100<br>0.94 (0.53-1.65)	0.110<br>0.45 (0.20-1.15)	0.200<br>0.52 (0.20-1.37)
**Additive**		0.300<br>1.30 (0.90-2.00)	0.070<br>0.66 (0.42-1.03)	0.272<br>0.80 (0.50-1.23)	0.474<br>1.19 (0.73-1.96)
		***KMT2A*-WT**			
***XRCC6* rs5751129**	**Control n (%)**	**ALL n (%)**		**AML n (%)**	
**TT**	118 (40.4)	19 (40.4)		17 (48.6)	
**TC**	133 (45.5)	21 (44.7)		15 (42.9)	
**CC**	41 (14.0)	7 (14.9)		3 (8.6)	
**Genetic Model**		***p*** **OR (CI)**	***p*** **aOR (CI)**	***p*** **OR (CI)**	***p*** **aOR (CI)**
**Dominant**		1.000<br>1.00 (0.53-2.00)	0.762<br>0.90 (0.47-1.71)	0.400<br>0.72 (0.40-1.45)	0.670<br>1.17 (0.56-2.43)
**Recessive**		0.824<br>1.10 (0.45-2.55)	0.638<br>0.80 (0.33-1.96)	0.600<br>0.60 (0.20-2.00)	0.532<br>1.49 (0.42-5.20)
**TT vs. TC**		1.000<br>1.00 (0.50-1.91)	0.852<br>0.93 (0.47-1.85)	0.575<br>0.80 (0.40-1.64)	0.810<br>1.09 (0.51-2.35)
**TT vs. CC**		1.000<br>1.10 (0.42-2.71)	0.504<br>0.71 (0.26-1.91)	0.411<br>0.51 (0.14-1.82)	0.592<br>1.43 (0.38-5.35)
**Additive**		1.000<br>1.01 (0.55-1.85)	0.699<br>0.88 (0.47-1.64)	0.300<br>0.70 (0.34-1.34)	0.587<br>1.21 (0.60-2.46)
		***KMT2A*-r**			
***XRCC6* rs5751129**	**Control n (%)**	**ALL n (%)**		**AML n (%)**	
**TT**	118 (40.4)	19 (32.2)		8 (44.4)	
**TC**	133 (45.5)	31 (52.5)		10 (55.6)	
**CC**	41 (14.0)	9 (15.3)		0 (0.0)	
**Genetic Model**		***p*** **OR (CI)**	***p*** **aOR (CI)**	***p*** **OR (CI)**	***p*** **aOR (CI)**
**Dominant**		0.310<br>1.43 (0.80-2.60)	0.100<br>1.67 (0.90-3.09)	0.810<br>0.85 (0.33-2.21)	0.536<br>0.73 (0.27-1.97)
**Recessive**		0.840<br>1.10 (0.50-2.41)	0.521<br>1.30 (0.57-2.95)	-	-
**TT vs. TC**		0.300<br>1.45 (0.80-2.70)	0.140<br>1.62 (0.85-3.08)	1.000<br>1.11 (0.42-2.90)	0.914<br>0.94 (0.34-2.56)
**TT vs. CC**		0.493<br>1.36 (0.57-3.25)	0.183<br>1.87 (0.74-4.75)	-	-
**Additive**		0.263<br>1.41 (0.80-2.52)	0.075<br>0.58 (0.31-1.05)	0.500<br>0.70 (0.30-1.80)	0.309<br>1.67 (0.61-4.53)

ALL: Acute Lymphoblastic Leukemia; AML: Acute Myeloid Leukemia; OR: Odds ratio; aOR: Odds ratio adjusted by non-whites and sex; *KMT2A*-WT: *KMT2A* wild type; *KMT2A*-r: *KMT2A* rearrangements.
