# Supplementary material for: XRCC4 rs28360071 intronic variant is associated with increased risk for infant acute lymphoblastic leukemia with KMT2A rearrangements
Source: Genet Mol Biol. 2020 Dec 2;43(4):e20200160. doi: 10.1590/1678-4685-GMB-2020-0160 (PMC7734917; doi:10.1590/1678-4685-GMB-2020-0160)
Supplement: Table S3 - [file 1415-4757-GMB-43-4-e20200160-s3.pdf]

Genetics and Molecular Biology
https://doi.org/10.1590/1678-4685-GMB-2020-0160

Supplementary Material to "*XRCC4* rs28360071 intronic variant is associated with increased risk for infant acute lymphoblastic leukemia with *KMT2A* rearrangements"

Table S3 - Genotype frequencies of *XRCC4* rs6869366 in EAL subtypes and *KMT2A* status.

***XRCC4* rs6869366**	**Control n (%)**	**ALL n (%)**		**AML n (%)**	
**TT**	249 (84.7)	97 (90.7)		75 (89.3)	
**TG**	43 (14.6)	10 (9.3)		7 (8.3)	
**GG**	2 (0.7)	0 (0.0)		2 (2.4)	
**Genetic Model**		***p*** **OR (CI)**	***p*** **aOR (CI)**	***p*** **OR (CI)**	***p*** **aOR (CI)**
**Dominant**		0.141<br>0.60 (0.30-1.20)	0.166<br>0.61 (0.35-1.10)	0.400<br>0.70 (0.31-1.42)	0.336<br>0.68 (0.31-1.47)
**Recessive**		-	-	0.215<br>3.60 (0.50-25.7)	0.195<br>3.76 (0.50-27.9)
**TT vs. TG**		0.185<br>0.59 (0.28-1.25)	0.207<br>0.60 (0.32-1.04)	0.200<br>0.54 (0.23-1.25)	0.180<br>0.55 (0.24-1.30)
**TT vs. GG**		-	-	0.240<br>3.32 (0.50-24.0)	0.234<br>3.38 (0.45-25.3)
**Additive**		0.107<br>0.54 (0.26-1.12)	0.133<br>1.75 (0.84-3.67)	0.610<br>0.80 (0.40-1.60)	0.540<br>1.25 (0.61-2.55)
***KMT2A*-WT**					
***XRCC4* rs6869366**	**Control n (%)**	**ALL n (%)**		**AML n (%)**	
**TT**	249 (84.7)	34 (85.0)		26 (89.7)	
**TG**	43 (14.6)	6 (15.0)		2 (6.9)	
**GG**	2 (0.7)	0 (0.0)		1 (3.4)	
**Genetic Model**		***p*** **OR (CI)**	***p*** **aOR (CI)**	***p*** **OR (CI)**	***p*** **aOR (CI)**
**Dominant**		1.000<br>1.00 (0.40-2.50)	0.947<br>1.03 (0.40-2.62)	0.600<br>0.70 (0.20-2.20)	0.496<br>1.54 (0.44-5.41)
**Recessive**		-	-	0.250<br>5.21 (0.50-59.3)	0.181<br>0.17 (0.01-2.28)
**TT vs. TG**		1.000<br>1.02 (0.41-2.60)	0.971<br>0.98 (0.38-2.50)	0.400<br>0.45 (0.10-1.95)	0.297<br>2.20 (0.49-9.75)
**TT vs. GG**		-	-	0.265<br>4.80 (0.42-54.6)	0.218<br>0.19 (0.01-2.61)
**Additive**		1.000<br>0.94 (0.40-2.35)	0.871<br>1.07 (0.42-2.74)	1.000<br>0.81 (0.30-2.44)	0.733<br>1.21 (0.39-3.72)
***KMT2A*-r**					
***XRCC4* rs6869366**	**Control n (%)**	**ALL n (%)**		**AML n (%)**	
**TT**	249 (84.7)	56 (94.9)		15 (78.9)	
**TG**	43 (14.6)	3 (5.1)		3 (15.8)	

https://doi.org/10.1590/1678-4685-GMB-2020-0160

***XRCC4* rs6869366**	**Control n (%)**	**ALL n (%)**		**AML n (%)**	
**GG**	2 (0.7)	0 (0.0)		1 (5.3)	
**Genetic Model**		***p* OR (CI)**	***p* aOR (CI)**	***p* OR (CI)**	***p* aOR (CI)**
**Dominant**		**0.040 0.30 (0.09-1.00)**	0.054 0.30 (0.08-1.01)	0.514 1.50 (0.50-4.65)	0.495 1.49 (0.46-4.76)
**Recessive**		-	-	0.172 8.11 (0.70-93.7)	0.082 10.6 (0.74-153.)
**TT vs. TG**		0.055 0.31 (0.10-1.04)	0.060 0.31 (0.09-1.05)	0.740 1.20 (0.32-4.20	0.810 0.85 (0.23-3.09)
**TT vs. GG**		-	-	0.170 8.30 (0.71-96.8)	0.103 9.16 (0.63-131.)
**Additive**		**0.040 0.30 (0.10-0.95)**	0.050 0.42 (0.01-1.11)	0.345 1.80 (0.61-5.10)	0.295 0.56 (0.19-1.64)

ALL: Acute Lymphoblastic Leukemia; AML: Acute Myeloid Leukemia; OR: Odds ratio; aOR: Odds ratio adjusted by skin color and sex; *KMT2A*-WT: *KMT2A* wild type; KMT2A-r: *KMT2A* rearranged.
