# Supplementary material for: XRCC4 rs28360071 intronic variant is associated with increased risk for infant acute lymphoblastic leukemia with KMT2A rearrangements
Source: Genet Mol Biol. 2020 Dec 2;43(4):e20200160. doi: 10.1590/1678-4685-GMB-2020-0160 (PMC7734917; doi:10.1590/1678-4685-GMB-2020-0160)
Supplement: Figure S1 - [file 1415-4757-GMB-43-4-e20200160-s4.pdf]

Genetics and Molecular Biology
https://doi.org/10.1590/1678-4685-GMB-2020-0160

Supplementary Material to "*XRCC4* rs28360071 intronic variant is associated with increased risk for infant acute lymphoblastic leukemia with *KMT2A* rearrangements"

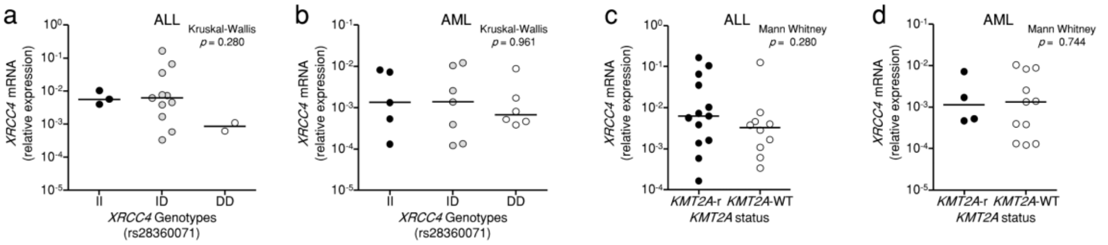

Figure S1 – Differential levels of *XRCC4* mRNA in EAL bone marrow samples. Expression levels of *XRCC4* in ALL patients (a) and AML patients (b) according to rs28360071 genotypes. Comparison of *XRCC4* expression levels in patients harboring *KMT2A*, rearranged (*KMT2A*-r) and *KMT2A*-WT according to rs28360071 genotypes in ALL patients (c) and AML patients (d). Experiments were performed in triplicates. Horizontal bars indicate median values.
