# Supplementary material for: XRCC4 rs28360071 intronic variant is associated with increased risk for infant acute lymphoblastic leukemia with KMT2A rearrangements
Source: Genet Mol Biol. 2020 Dec 2;43(4):e20200160. doi: 10.1590/1678-4685-GMB-2020-0160 (PMC7734917; doi:10.1590/1678-4685-GMB-2020-0160)
Supplement: Figure S2 - [file 1415-4757-GMB-43-4-e20200160-s5.pdf]

Genetics and Molecular Biology
https://doi.org/10.1590/1678-4685-GMB-2020-0160

Supplementary Material to "*XRCC4* rs28360071 intronic variant is associated with increased risk for infant acute lymphoblastic leukemia with *KMT2A* rearrangements"

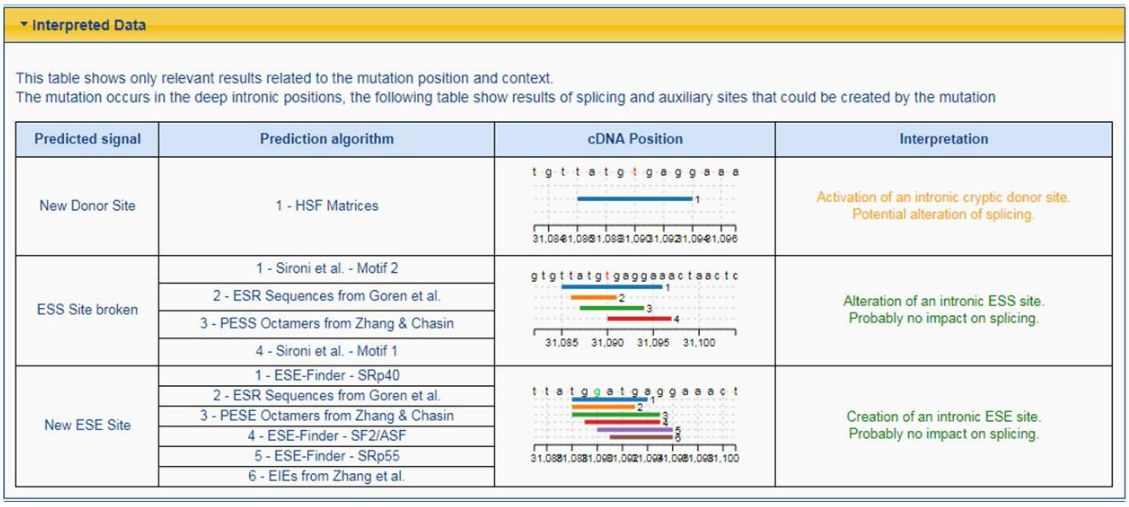

▾ Interpreted Data

This table shows only relevant results related to the mutation position and context.
The mutation occurs in the deep intronic positions, the following table show results of splicing and auxiliary sites that could be created by the mutation

Predicted signal	Prediction algorithm	cDNA Position	Interpretation
New Donor Site	1 - HSF Matrices	t g t t a t g t g a g g a a a	Activation of an intronic cryptic donor site. Potential alteration of splicing.
ESS Site broken	1 - Sironi et al. - Motif 2	g t g t t a t g t g a g g a a a c t a a c t c<br>31,085 31,090 31,095 31,100	Alteration of an intronic ESS site. Probably no impact on splicing.
	2 - ESR Sequences from Goren et al.		
	3 - PESS Octamers from Zhang & Chasin		
	4 - Sironi et al. - Motif 1		
New ESE Site	1 - ESE-Finder - SRp40	t t a t g g a t g a g g a a a c t	Creation of an intronic ESE site. Probably no impact on splicing.
	2 - ESR Sequences from Goren et al.		
	3 - PESE Octamers from Zhang & Chasin		
	4 - ESE-Finder - SF2/ASF		
	5 - ESE-Finder - SRp55		
	6 - EIEs from Zhang et al.		

Figure S2 – *In silico* predictions for *XRCC4* rs28360071 polymorphism (NM_022550.3:c.315+31090del30) using Human Splicing Finder software version 3.1.
