# Supplementary material for: XRCC4 rs28360071 intronic variant is associated with increased risk for infant acute lymphoblastic leukemia with KMT2A rearrangements
Source: Genet Mol Biol. 2020 Dec 2;43(4):e20200160. doi: 10.1590/1678-4685-GMB-2020-0160 (PMC7734917; doi:10.1590/1678-4685-GMB-2020-0160)
Supplement: Figure S3 - [file 1415-4757-GMB-43-4-e20200160-s6.pdf]

Genetics and Molecular Biology
https://doi.org/10.1590/1678-4685-GMB-2020-0160

Supplementary Material to "*XRCC4* rs28360071 intronic variant is associated with increased risk for infant acute lymphoblastic leukemia with *KMT2A* rearrangements"

a

```
309 GAGCAGGACC AGCTGATGTA TACACGTTTA ATTTTTCTAA AGAGTCTTGT
359 TATTTCTTCT TTGAGAAAAA CCTGAAAGAT GTCTCATTCA GACTTGGTTC
409 CTTCAACCTA GAGAAAGTTG AAAACCCAGC TGAAGTCATT AGAGAACTTA
459 TTTGTTATTG CTTGGACACC ATTGCAGAAA ATCAA
```

b

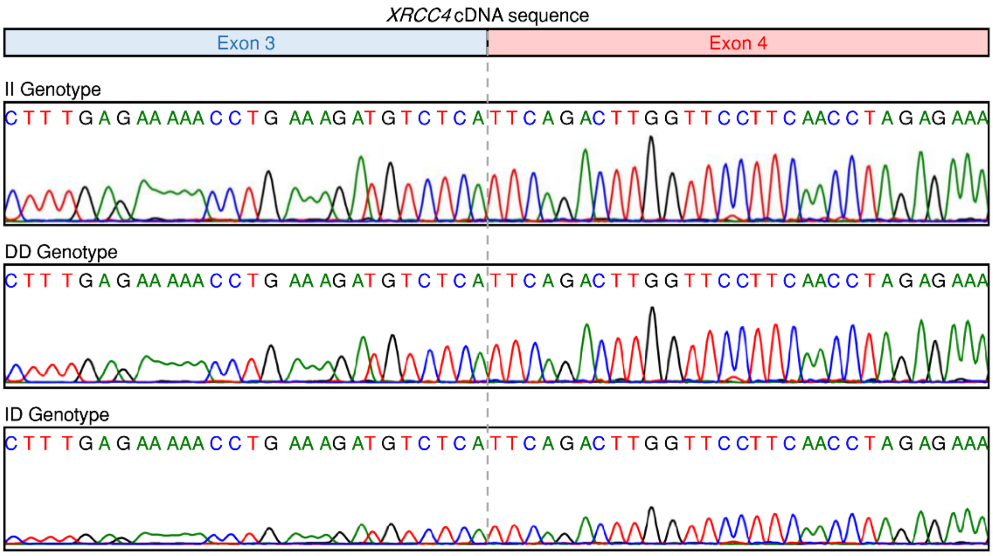

Figure S3 - Sequencing of bone marrow *XRCC4* cDNA of patients with EAL. (a) Sequence of exon 3 and exon 4 in blue and red, respectively. Annealing sequences of primers are underlined. (b) Representative chromatograms of selected patients harboring the three different genotypes of rs28360071.
